# The Neuro-Glial Properties of Adipose-Derived Adult Stromal (ADAS) Cells Are Not Regulated by Notch 1 and Are Not Derived from Neural Crest Lineage

**DOI:** 10.1371/journal.pone.0001453

**Published:** 2008-01-16

**Authors:** Philip C. Wrage, Thi Tran, Khai To, Edward W. Keefer, Kelly A. Ruhn, John Hong, Supriya Hattangadi, Isaac Treviño, Malú G. Tansey

**Affiliations:** 1 Department of Physiology, The University of Texas Southwestern Medical Center, Dallas, Texas, United States of America; 2 Department of Plastic Surgery, The University of Texas Southwestern Medical Center, Dallas, Texas, United States of America; National Institutes of Health, United States of America

## Abstract

We investigated whether adipose-derived adult stromal (ADAS) are of neural crest origin and the extent to which Notch 1 regulates their growth and differentiation. Mouse ADAS cells cultured in media formulated for neural stem cells (NSC) displayed limited capacity for self-renewal, clonogenicity, and neurosphere formation compared to NSC from the subventricular zone in the hippocampus. Although ADAS cells expressed Nestin, GFAP, NSE and Tuj1 *in vitro*, exposure to NSC differentiation supplements did not induce mature neuronal marker expression. In contrast, in mesenchymal stem cell (MSC) media, ADAS cells retained their ability to proliferate and differentiate beyond 20 passages and expressed high levels of Nestin. In neuritizing cocktails, ADAS cells extended processes, downregulated Nestin expression, and displayed depolarization-induced Ca^2+^ transients but no spontaneous or evoked neural network activity on Multi-Electrode Arrays. Deletion of Notch 1 in ADAS cell cultures grown in NSC proliferation medium did not significantly alter their proliferative potential *in vitro* or the differentiation-induced downregulation of Nestin. Co-culture of ADAS cells with fibroblasts that stably expressed the Notch ligand Jagged 1 or overexpression of the Notch intracellular domain (NICD) did not alter ADAS cell growth, morphology, or cellular marker expression. ADAS cells did not display robust expression of neural crest transcription factors or genes (Sox, CRABP2, and TH); and lineage tracing analyses using Wnt1–Cre;Rosa26R-lacZ or -EYFP reporter mice confirmed that fewer than 2% of the ADAS cell population derived from a Wnt1-positive population during development. In summary, although media formulations optimized for MSCs or NSCs enable expansion of mouse ADAS cells *in vitro,* we find no evidence that these cells are of neural crest origin, that they can undergo robust terminal differentiation into functionally mature neurons, and that Notch 1 is likely to be a key regulator of their cellular and molecular characteristics.

## Introduction

Neurodegenerative diseases are difficult to treat because damaged neurons in the central nervous system (CNS) have a limited capacity for regeneration and repair compared to neurons in the peripheral nervous system (PNS) or tissues in other organs (e.g. liver). Therefore, efforts to identify suitable tissue sources to replace lost neuronal populations or aid in the regeneration of CNS pathways has intensified. The use of embryonic stem (ES) cells for clinical applications is limited at present by the low efficiency with which these can be differentiated into functional neurons *in vitro*, the tumorigenic potential of ES cells that escape differentiation, and the need for immunosuppression of the host. In theory, neural stem cells would be the ideal choice for cell replacement, but they are limited in number and difficult to access for autologous cell transplantation.

It has been suggested that the readily accessible adipose-derived adult stromal (ADAS) cells of mesenchymal origin may represent a source of adult stem cells with neurogenic potential because they have the capacity to extend neuritic processes and express protein markers characteristic of neural stem cells *in vitro* in response to neuritizing cocktails [1]–[5]. Although ADAS cells have been shown to display the potential for multilineage specification, reports on their ability to demonstrate clonogenic potential, undergo unlimited self-renewal, and become terminally differentiated into mature neurons are in conflict (reviewed in [6]) and must be critically re-examined in order to gain insight into their biology and evaluate their potential value in cell replacement therapies.

The Notch 1 pathway is a master regulator of neurogenesis in the CNS [7]–[9] and maintains ‘stemness’ in the neuroglial progenitor pool [7] while regulating glial differentiation later in development [8], [10]–[12]. In neural crest stem cells, Notch 1 signaling suppreses neurogenesis and promotes Schwann cell development [8], [13], [14]. Given this well-documented role of Notch 1 in regulating proliferative capacity, cell fate acquisition, and differentiation of neural stem cell populations, we investigated the effect of Notch 1 deletion on mouse ADAS cell proliferation and expression of neural markers *in vitro*.

Lastly, expression of tyrosine hydroxylase (TH), choline acetyltransferase (ChAT), GABA, and serotonin (5-HT) by ADAS cells in culture [3], [5] raise the interesting possibility that the neurogenic properties of ADAS cells may be derived from their neural crest origin. During development, the overlying ectoderm gives rise to neural crest cells which delaminate, migrate to the periphery, and give rise to a number of different tissues (reviewed in [15]) with demonstrated neurogenic potential *in vitro,* including skin-derived precursors (SKPs) [16]. Therefore, we hypothesized that ADAS cells may harbor a population of neural crest derived stem cells that has remained resident in adipose tissue into adulthood.

## Materials and Methods

### Animals

Animal maintenance and experimental procedures were in accordance with the NIH Guidelines for Animal Care and Use and approved by the Institutional Animal Care and Use Committee (IACUC) at The University of Texas Southwestern Medical Center. Animals were housed in a climate controlled facility staffed with certified veterinarians. Wnt1-Cre reporter mice were obtained from Jackson Labs (Bar Harbor, ME). This strain originated on a B6CBA F1/J background whereupon the founder animals were mated with Swiss out-bred mice to homozygosity. Homozygous males were crossed with females of one of two reporter lines that were maintained on a B6 background: Rosa26R-lacZ [17] or Rosa26R-EYFP [18] reporter mice to generate heterozygous compound transgenic reporter mice. To ensure that adipose tissue for ADAS cell cultures was harvested from heterozygous mice, Cre reporter mouse strains were genotyped by PCR using published primers [17]: AAA GTC GCT CTG AGT TGT TAT; GCG AAG AGT TTG TCC TCA ACC; GGA GCG GGA GAA ATG GAT ATG.

### Neural Stem Cell Harvest and Culture

Mouse neural stem cells for neurosphere cultures were obtained from the frontal cortex of mouse pups (P2–P6). Briefly, following incubation in DDP [(DNase I (1 U/mL), Dispase II (1.2 U/mL), Papain (20 mg/mL)] for 30 min, minced tissue was filtered, centrifuged and resuspended in Neural Stem Cell Media [NSCM, DMEM/F12 with N2 Supplement, 2 mM L-glutamine, 1mM sodium pyruvate, penicillin/streptomycin (10 U/mL and 0.01 mg/mL), 20 ng/mL EGF, 10 ng/mL FGF-2] or Medium A. Trypan blue exclusion was used to assess cell viability and 1×10^6^ cells were plated in a 10 cm dish in 5–10 mL of NSCM or Medium A.

### Tissue Harvesting and ADAS Cell Culture

Animals were euthanized with halothane prior to decapitation. Intrascapular, inguinal, and abdominal fat pads were removed from mice into sterile phosphate-buffered saline (PBS, pH 7.4), rinsed, and transferred to Dulbecco's Modified Eagle Medium (DMEM) containing 10% fetal bovine serum (FBS) and antibiotic/antimycotic (Gemini) for a 1-hour incubation at 37°C at 5% CO_2_. Fat pads were then minced, and digested in serum-free DMEM containing 0.075% type-I collagenase (Invitrogen) for 1 hour (with vortexing every 10 min) at 37°C. Collagenase was neutralized with an equal volume of DMEM/10% FBS and minced tissue was centrifuged at 1000×g for 4 minutes at room temperature. The supernatant containing mature adipocytes was removed by aspiration; the pellet containing the stromal-vascular fraction was resuspended in serum-free DMEM. Following a second centrifugation step (1000×g for 4 minutes), cells were filtered through a Nytex filter and plated in 2 mL of one of the following growth media into a 35 mm^2^ cell culture dish: mouse ADAS cells were grown in Medium A (NeuroCult Proliferation Medium), Medium B ([DMEM/F12, 100 U penicillin, 100 µg streptomycin, 2 mM L-glutamine, 1× N2 Supplement, EGF (10 ng/mL; R&D), FGF-2 (20 ng/mL; R&D), and mLIF (10 ng/mL; R&D)], or Medium C ([MesenCult Basal Media (Stem Cell Technologies) supplemented with MesenCult Proliferation Supplement (1∶5), 100 U penicillin and 100 µg streptomycin). Non-adherent cells in P0 cultures were removed 24 hrs post-plating to expand the small number of adherent stromal cells (<5% of total cells plated) by serial passage at ratios of 1∶3 or 1∶4. All cells used for experiments were between P2 and P4.

ADAS cell cultures from rat were established from a 0.75 cm^2^ portion of the interscapular fat pad of adult female Sprague-Dawley rats (220–230 g). The adipose tissue was dissociated and processed as indicated above. The final cell pellet was resuspended in DMEM/10% FBS (D10 medium) and plated (Day 0, P0) onto 35 mm tissue culture-treated dishes. Non-adherent cells in P0 cultures were removed 24 hrs post-plating to expand the small number of adherent stromal cells (<5% of total cells plated) by serial passage at ratios of 1∶3 or 1∶4. All cells used for experiments were between P2 and P4.

### Clonogenic Analysis of ADAS Cells

ADAS cells were plated at clonal densities (2.5×10^3^ cells/mL) in Medium A in a 35 mm^2^ cell culture overlaid with collagen-based matrix dishes as per manufacturer's instructions (Stem Cell Technologies). Cultures were fed twice weekly and periodically examined for colony formation for 3–4 weeks. For quantitation, phase-contrast images were taken with a 10× objective and 10 fields per well were counted. Results are expressed as the mean±S.E.M.

### Neural Induction of ADAS Cell Cultures

To induce neural differentiation of mouse ADAS cells, we modified published protocols based on N2 medium supplemented with valproic acid or retinoic acid/forskolin (Hsieh et al., 2004) or Neural Induction Medium (Safford et al., 2004). Differentiation of rat ADAS cell cultures (P2-P4) consisted of pre-induction of cultures plated in D10 with 10 ng/ml EGF and 20 ng/ml bFGF for 2 to 3 days prior to a 4-day exposure to either of the following: Neural Differentiation Medium (NDM: Dulbecco's Modified Eagle's Medium+10% FBS, 120 uM indomethacin, 3 ug/mL insulin, 300 uM isobutyl-methylxanthine)

#### FACS analysis of ADAS cultures

Quantitative FACS analysis of EYPF-positive cells from ADAS cell cultures grown in Mesencult derived from Wnt1-Cre;Rosa26R-stop-EYFP compound transgenic mice were performed using a BD FACSCalibur system with a 530 nm GFP filter and an FL2 565-605 nm filter to correct for autofluorescence.

### Nucleofection of ADAS cells

To overexpress levels of the Notch intracellular domain (NICD), rat ADAS cells (1×10^6^ cells in 100 ul of Cell Line T Nucleofector solution) were nucleofected with a) 2 ug of a pCS2 plasmid encoding a version of constitutively activated membrane-bound Notch-1 (NDeltaE) described previously [19] and kindly provided to us by Dr. Rafi Kopan at Washington University and 1ug of pMAX plasmid encoding Enhanced Green Fluorescence Protein (EGFP) provided by the manufacturer, or b) 2 ug of pMAX EGFP plasmid, or c) no vector. As per the manufacturer's instructions, optimization experiments were conducted and Amaxa program T-20 was selected for experiments on the basis of pilot studies to maximize transfection efficiency and cell viability. Immediately after nucleofection, cells were placed in pre-warmed DMEM/10% FBS and placed in a 37°C incubator with a humidified 95% O_2_/5% CO_2_ atmosphere. Cell viability after nucleofection ranged from 70–90%. Two days later, nucleofected cells were treated with 10 ng/mL EGF and 20 ng/mL FGF-2 for 24 hrs followed by 2 additional days in NDM.

### Co-culture of ADAS cells with Jagged-1-expressing Fibroblasts

Rat ADAS cells grown in DMEM/10%FBS were co-cultured at a 1∶1 ratio with an L fibroblast cell line (SN3T9) that stably expresses Jagged-1 [20] kindly provided to us by Dr. Gerry Weinmaster at UCLA.

### Fluo-4 AM Calcium Measurements

ADAS cells were incubated with the cell-permeant calcium indicator dye Fluo-4 AM (3 µm) (Molecular Probes/Invitrogen) for 45 minutes at 37°C in a humidified 95% O_2_/5% CO_2_ atmosphere then incubated in phenol red free, serum free media for 15–30 minutes at 37°C before imaging.

### Electrophysiological field recordings of ADAS Cell Cultures on Multi-Electrode Arrays

Monolayers of ADAS cell cultures grown on dual-network Multi-Electrode Arrays (MEA) (2×32 electrodes) were placed in a stainless steel static bath recording chamber containing two 2 ml reservoirs, allowing physical isolation of the cultures. Electrophysiological recordings were performed using a Plexon Inc. (Dallas) 64-channel recording system; electrical stimulation was delivered via a Multichannel Systems MED-4 stimulator (Reutlingen, Germany). To examine evoked activity, cultures were electrically stimulated with voltage-controlled 0.75 V biphasic pulses (positive phase first) consisting of 5 pulses at 50 Hz repeated every 30 seconds for 150 seconds (total of 30 pulses). Each channel was stimulated sequentially (64 stimulus trials per MEA, 1 trial per channel). Evoked responses were also examined pharmacologically with sequential bath application of glutamate (5 µM) and the GABA-A receptor antagonist bicuculline (20 µM) 30 minutes later. Experiments were performed at 37°C in a humidified 5% CO2 atmosphere.

### Immunocytochemistry

ADAS cells were plated at 1-2×10^4^ cells per well in 4-well dishes and grown in Medium A, B, or C for immunocytochemical analyses of neuro-glial marker expression before and after incubation in the specified neural induction cocktails. Neurosphere-like clusters formed in P1 or P2 cultures were dissociated into single cells before plating by mechanical trituration with a fire-polished pipette and limited trypsin (0.25%) exposure. Half of the ADAS cells were cultured for up to one week in Medium A or B in the 4-well dishes and fixed with 4% paraformaldehyde (PFA) in PBS when they reached ∼50% confluence. The remainder of the ADAS cells were treated for 48 hours with NeuroCult Differentiation Media prior to fixation with 4% PFA. Nestin and Ki67 antibodies were obtained from BD Pharmingen, NSE from Polysciences, Tuj1, MAP2b, GAP-43, and GFAP from Chemicon. Alexa-488 or Alexa-594 conjugated secondary antibodies (Invitrogen) diluted 1∶1000 were used for immunofluorescence detection of desired antigens. Cell nuclei were visualized with the nuclear dye Hoechst 33258 (bis-benzimide). A CoolSnap ES monochrome camera mounted on an upright Olympus BX61 or an inverted Olympus CK40 fluorescence scope was used for image capture and MetaMorph software was used for image analyses.

#### Lentiviral transduction

Lentiviruses encoding Cre recombinase or mutant Cre recombinase were a generous gift from Dr. Thomas Sudhof. The lentiviral supernatants were obtained by transfecting 293 HEK cells with a plasmid encoding Cre recombinase or mutant (Delta) Cre recombinase plus the appropriate packaging plasmids. Lentiviral titers used for experiments were 1×10^9^ infectious units/mL and the efficiency of ADAS cell transduction was generally greater than 90%.

#### Quantification of Nestin and Tuj-1Expression in Notch1-deleted ADAS cell cultures

To estimate the extent to which exposure to NeuroCult Differentiation Medium induced downregulation of Nestin and Tuj-1 expression in ADAS cells transduced with lenti-Cre or lenti-mutant-Cre, optical density of neural marker immunoreactivity was measured using the Image-Pro Plus 5.1. Integrated optical density readings were corrected for nonspecific background density, as measured on cells stained with non-immune IgG serum. Images were taken under a 20× objective and converted to gray scale; six different fields were surveyed throughout the well in triplicate wells, subsequently averaged for each treatment condition. Mean and S.E.M were calculated for each group. Inter-group differences between the various dependent variables were assessed using one-way ANOVA, followed by the Tukey-Kramer post hoc multiple comparisons test. Data obtained were analyzed by GraphPad statistic software; p<0.05 were considered significant.

### Real-Time Quantitative PCR (QPCR)

Real-time quantitative PCR (QPCR) was performed as previously described [21]. Briefly, total RNA was isolated from cultured cells or rat tissues, treated with DNase I, and reverse transcribed using Superscript II RNase H- reverse transcriptase. Quantitative real-time PCR was performed using an ABI Prism 7000 Detection System (Applied Biosystems). Each reaction was performed in a volume of 20 µl that contained 50 ng cDNA, 10 µl SYBR green PCR Master Mix, and 150 nM of each PCR primer. All reactions were performed in triplicate. Levels of various mRNAs were normalized to those of the mouse house-keeping gene cyclophilin. Oligonucleotide primers for QPCR were obtained from Integrated DNA Technologies (Coralville, IA). The following mouse primers sequences were used for gene amplification: Nestin: forward 5′-GGT CAC TGT CGC CGC TACT C-3′ and reverse 5′-CGG ACG TGG AGC ACT AGA GAA–3′; Neuron Specific Enolase (NSE): forward 5′-TGA TCT TGT CGT CGG ACT GTG T–3′ and reverse 5′-CTT CGC CAG ACG TTC AGA TCT-3′; Glial-Fibrillary Acidic Protein (GFAP): forward 5′-TGG AGG GCG AAG AAA-3′ and reverse 5′-CGG TGG AGG TTG GAG AA-3′; Tuj-1: forward 5′-GGT CTG GCG CCT TTG GA–3′ and reverse 5′-CAC CAC TCT GAC CAA AGA TAA AGT TG-3′; Sox 9: forward 5′-CAG TAC CCG CAT CTG CAC AA–3′ and reverse 5′-CCT CCA CGA AGG GTC TCT TCT–3′; for tyrosine hydroxylase (TH) forward 5′-TGT TGG CTG ACC GCA CAT T-3′ and reverse 5′-GCC CCC AGA GAT GCA AGT C–3′; for CRABP2 forward 5′-CCT CCT GGA GCC GAG AAC T-3′ and reverse 5′-ACA CAA CGT CAT CTG CTG TCA TT–3′; for Notch 1 forward 5′-ATT GAA AGC ACA TAT GGA GAT-3′ and reverse 5′-GTA TAA GCA TGA AGT GGT CCA-3′.

### Statistical Analyses

Results are expressed as the mean±S.E.M of two to three experiments with treatments in duplicate. Statistical significance was assessed with an analysis of variance (ANOVA) followed by Bonferroni *t-*test using Prism GraphPad. A value of *p*<0.05 was considered significant.

## Results

### Proliferation of mouse ADAS cells in Neural Stem Cell Media Formulations

**Figure 1:**
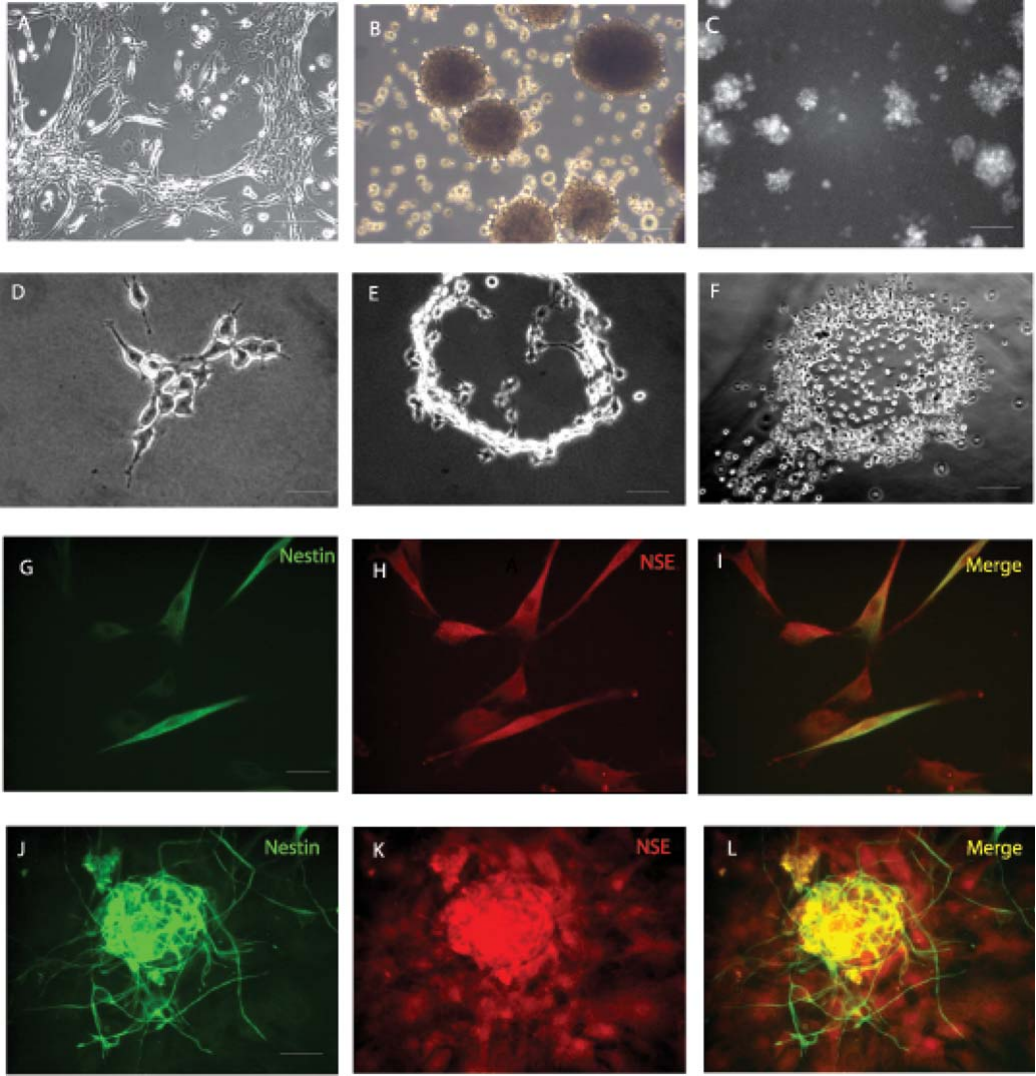
Proliferation of mouse adipose-derived adult stromal (ADAS) cells in media formulations optimized for neural stem cells. Cells grow as adherent cultures (A) and upon reaching high confluence they form neurosphere-like clusters (B) in a serum-free media formulation (Media A) which are indistinguishable from neurospheres derived from the subventricular zone in the hippocampus of young adult mice (C); Single-cell derived colonies growing in NeuroCult (Media B) (D) expand to form ring-like structures (E, F); Neural stem cell marker expression in adherent mouse ADAS cells (G–I) and neurospheres (J–L) proliferating in Media A. NSE, Neuron Specific Enolase. Scale bar in A, B, C and F = 220 um; scale bar in D, E G–I = 55 um; scale bar in J–L = 110 um.

**Figure 2:**
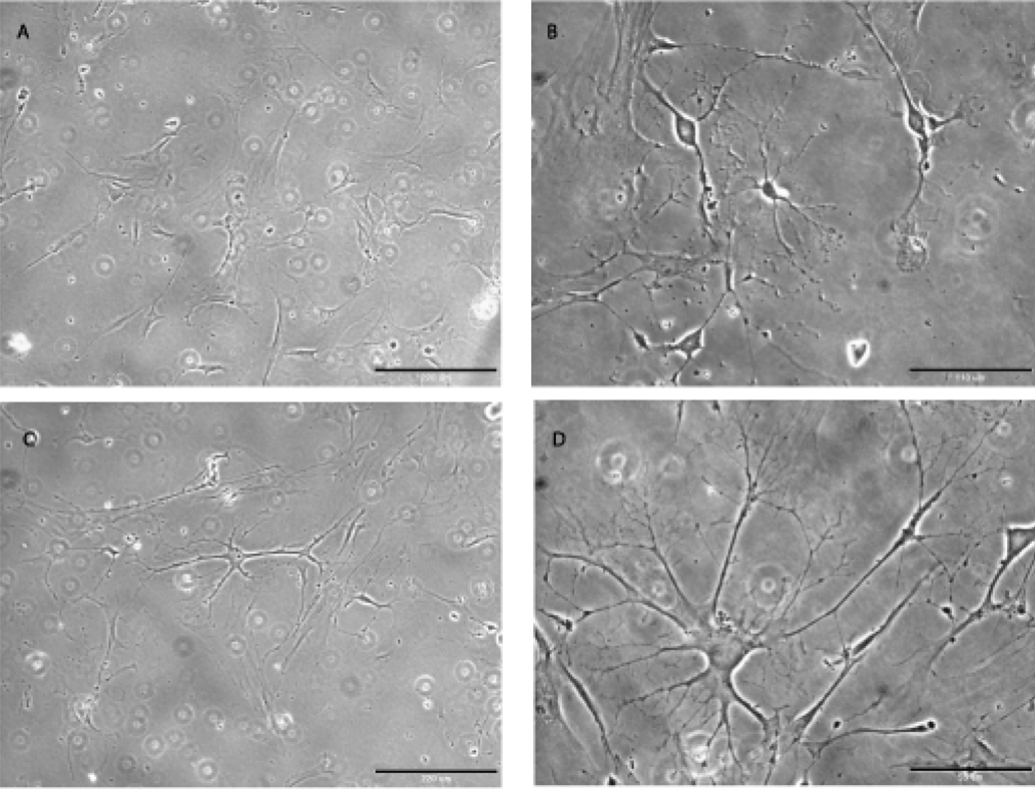
Proliferation and differentiation of mouse ADAS cells in cell culture medium designed for mesenchymal stem cells. Phase-contrast image of undifferentiated mouse ADAS cells at passage 5 (A) and passage 22 (C); process outgrowth in the same cells after 4-hr exposure to Neural Induction Medium (NIM)(B and D, respectively). Scale bar in A and C = 220 um; scale bar in B = 110 um; scale bar in D = 55 um.

Reports indicating that ADAS cell cultures could be coaxed to express early neuronal markers *in vitro* with low efficiency led us to investigate whether media formulations specifically designed for neural stem cells (NSCs) could be used to expand these cells *in vitro* for further characterization. We identified two formulations optimized for expansion of NSCs in which ADAS cell monolayers could be maintained in culture. In addition, adherent cells **(** **Figure 1A** **)** grown to confluence were able to cluster and form neurosphere-like clusters which eventually detached and continued to proliferate in suspension occasionally giving rise to secondary and tertiary neurospheres **(** **Figure 1B** **).** These neurosphere-like clusters were visually indistinguishable from those formed by NSCs derived from the subventricular zone of post-natal day 3 (P3) Nestin-GFP transgenic mouse pups (courtesy of Steven Kernie) which also grew in suspension as neurospheres **(** **Figure 1C** **)**. Mechanical dissociation enabled us to subculture adherent monolayers in formulations optimized for NSCs neurospheres. Unlike culture in Medium A or B which only supported growth of ADAS cells and neurosphere formation for three passages, culture of ADAS cells in the MSC-optimized Medium C supported subculturing of ADAS well beyond 5 passages **(** **Figure 2** **).**

### Limited Clonogenic potential of mouse ADAS cells in NeuroCult Medium

To investigate whether mouse ADAS cells had the potential to form clonal colonies from single cells, we performed clonogenic analyses on ADAS cells grown under media formulations optimized for culture of NSCs. Most of the colonies **(** **Figure 1D** **)** that formed from single cells 3 to 4 weeks after plating resembled rings **(** **Figure 1E, F** **)**, and none of them had the condensed, tightly packed, spherical architecture characteristic of true adult NSCs. In contrast with published reports [1], [3], our results indicate that ADAS cells have a limited capacity to form clonal colonies from single cells in media formulations optimized for neural stem cell culture; yet it was of interest to confirm whether expression of neural and glial markers could be achieved in ADAS cells grown in NSC-optimized media under basal conditions or after exposure to neuronal differentiation cocktails.

### Expression of Neural Stem Cell Markers in ADAS cells grown in Neural Stem Cell Media Formulations

We investigated whether mouse ADAS cells could adopt molecular and cellular characteristics of neural stem cells when proliferated in media formulations optimized for NSCs and after exposure to neural induction cocktails. Immunocytochemical analyses with antibodies specific for neural stem cell markers including Nestin, glial fibrillary acidid protein (GFAP), neuron specific enolase (NSE), and beta-tubulin III (Tuj1) indicated robust expression of several of these markers in these cells at all passages **(** **Figure 1G–L** **and Supplemental Table S1)**. We observed no significant outgrowth of processes or any other morphological differentiation into neuron-like cells after exposure to NeuroCult Differentiation Media for up to 96 hrs. However, after 48 hr treatment of ADAS cells with NeuroCult Differentiation Media, the fraction of cells positive for Nestin, NSE and Tuj1 immunoreactivity was decreased while the occasional MAP2b-immunoreactive cell appeared. GFAP expression remained relatively high and detectable before and after exposure to NeuroCult Differentiation Media. In summary, media formulations designed for NSCs and known to induce neuronal differentiation of NSCs promoted expression of neural and glial markers in ADAS cells even though no significant morphological differentiation was observed (e.g., extension of processes). Given the limited ability to passage these cells in the media formulations optimized for NSCs, we investigated whether ADAS cells could be expanded in formulations designed for mesenchymal stem cells (MSCs).

### Proliferation of mouse ADAS cells in Mesenchymal Stem Cell Media Formulations

Flow cytometric analyses of ADAS cells have demonstrated that these cells express many of the cell surface markers expressed by MSCs including CD29, CD44, CD105, and CD 166 [4], [22]. Therefore, we investigated the ability of ADAS cells to grow in media specifically formulated for the enrichment and culture of MSCs. We found that ADAS cells grown in Media C (MesenCult) retained high viability and proliferated for at least 22 passages **(** **Figure 2** **)**. These findings are consistent with the possibility that ADAS cells represent a mesoderm-derived stromal cell population which may contain resident adult MSCs that can be expanded *in vitro.*

### Morphological Differentiation of ADAS cells grown in MesenCult and exposed to Neural Induction Medium

Cell morphology of ADAS cells grown in Media C (MesenCult) changed rapidly (within 1–4 hours) after exposure to neural induction paradigms known to induce differentiation of adult neural stem cells [23] or human ADAS cells [5]. Specifically, we observed development of cytoplasmic extensions and appearance of phase-bright cell bodies with a large nucleus-to-cytoplasm ratio **(** **Figure 2** **).** Neural induction with RA/forskolin yielded similar results (data not shown). Because similar observations reported for chemically-induced neural differentiation of bone marrow-derived stromal cells have been ascribed to cellular toxicity, cell shrinkage, and changes in the cytoskeleton rather than to regulated steps in a cellular differentiation program [24], it was important to determine whether morphological differentiation of ADAS cells grown in Media C into neuron-like cells in response to neuritizing cocktails was accompanied by the expression of protein markers characteristic of neural and glial progenitor populations.

### Expression of Neural Stem Cell Markers in ADAS cells grown in Mesenchymal Stem Cell Media

**Figure 3:**
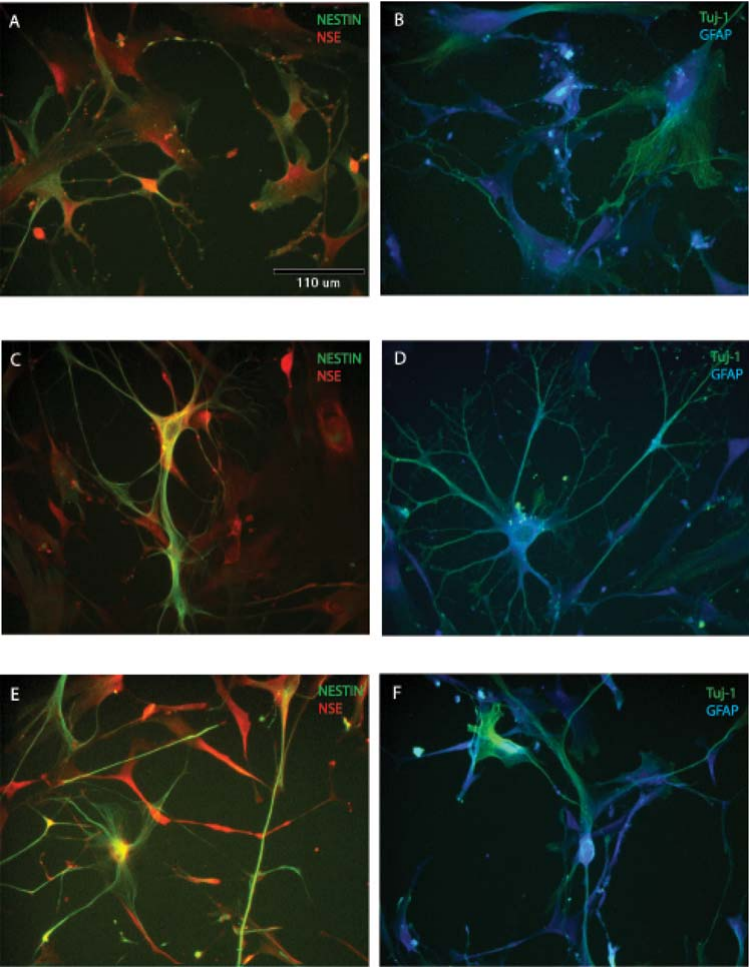
Expression of neural stem cell and neuroblast markers in mouse ADAS cells grown in MesenCult (Media C). Expression of neural markers in MesenCult proliferation media (A, B) and after 4-hr (C, D) or 24-hr (E, F) exposure to Neural Induction Medium (NIM). NSE, Neuron Specific Enolase; GFAP, glial-fibrillary acidic protein; Tuj1, β-tubulin III. Scale bar in A–F = 110 um.

Immunocytochemical analyses indicated that expression of neural stem cell markers under basal conditions in undifferentiated ADAS cells expanded *in vitro* in Medium C was extremely low except for that of Nestin **(** **Table 1** **).** Following treatment with neural induction protocols, ADAS cells up-regulated expression of Nestin, Neuron Specific Enolase (NSE), glial fibrillary acidic protein (GFAP), Tuj-1 (β-tubulin III) detectable by immunocytochemistry and verified by real-time QPCR within 4 hours of exposure to NIM that persisted for 24 hours following neural induction **(** **Figure 3** **and** **Table 1** **).** Beyond 48 hour exposure to NIM, down-regulation of Nestin and to a lesser extent NSE and Tuj-1 correlated with the appearance of MAP2b and GAP43-expressing cells while levels of GFAP were maintained.

**Table 1:** Expression of neural stem cell markers in mouse ADAS grown in Mesencult and exposed to Neural Induction Medium (NIM).

Marker	Baseline	4 hr NIM	24 hr NIM	48 hr NIM
Nestin	++	+++	++	+
NSE*	+/−	++	+++	+++
GFAP*	+/−	+++	+++	++
Tuj1*	+	++	+++	++

Mouse ADAS cells were proliferated in Mesencult and exposed to NIM for the times indicated prior to harvest in 4% paraformaldehyde for immunocytochemical analyses.

Semi-quantitative assessment of marker expression was performed using the following criteria: +++ = robust expression in greater than 50% of the cells; ++ = moderate expression in greater than 50% of the cells; + = low expression in more than 50% of the cells; +/− = low expression in less than 50% of the cells; − = no detectable expression. Asterisk (*) indicates marker expression confirmed by real-time PCR. Abbreviations: NIM, Neural Induction Medium; NSC, Neural Stem Cell; NSE, Neuron Specific Enolase; GFAP, Glial fibrillary acidic protein; Tuj1, β-tubulin III

Taken together, our multiple analyses suggest cell culture conditions optimized for mesenchymal stem cells support expansion of mouse ADAS cells and retention of molecular markers characteristic of neuro-glial progenitors. To further explore the mechanisms by which the neuron-glial characteristics of ADAS cells were regulated, we investigated the role of signaling pathways known to regulate growth and differentiation of progenitor pools and neuronal fate acquisition.

### The role of Notch 1 in ADAS cell proliferation and expression of neural stem cell markers

**Figure 4:**
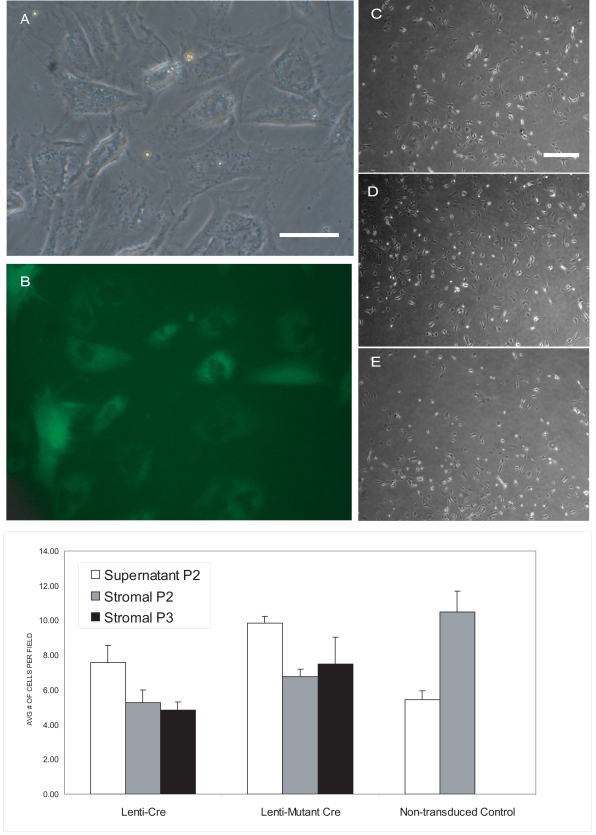
Notch 1 deletion has minimal effects on ADAS cell proliferation. Transduction efficiency in mouse ADAS cells with lentiviral-GFP (A,B); Proliferation of floxed Notch1 ADAS cells transduced with lentiviral-Cre recombinase (C), mutant Cre (D), or no virus (E); Quantification of the effect of Notch 1 deletion on clonogenic potential of ADAS spheres from the supernatant or stromal fraction (F). Scale bar in A and B = 55 um; scale bar in C–E = 220 um.

The Cre-lox system has been effectively used to conditionally delete a wide variety of genes *in vitro* and *in vivo.* ADAS cell cultures established from adipose tissue of mice harboring a floxed-Notch 1 allele allowed us to ask whether endogenous Notch 1 signaling has an important role in regulating ADAS cell proliferation and differentiation. ADAS cell cultures were established from fat pads isolated from mice harboring a floxed Notch 1 (fN1) allele. No significant difference in gross morphology or proliferation kinetics was observed between wild type and fN1 mouse ADAS cells (data not shown). Upon confluence of the initial cell plating (P0), neurosphere-like cells as well as adherent cells were passaged (P1) and maintained in culture as separate populations. Neurosphere-like clusters of cells floating in the supernatant and cells derived from the adherent stromal faction were transduced with lentivirus vectors (See Methods) driving expression of wild-type Cre recombinase or a catalytically inactive mutant Cre (delta Cre). Both expression constructs contained an internal ribosome entry site (IRES) upstream of an EGFP gene allowing for convenient identification of transduced cells using green fluorescence. Formation of secondary and tertiary neurosphere-like clusters in fN1 ADAS cells infected with lenti-Cre, lenti-mutant Cre, or mock-infected was monitored for several passages and found to be the same under all experimental conditions (data not shown). Neurosphere-like clusters were dissociated into a single-cell suspension and FACS-sorted based on GFP fluorescence. GFP–negative cells were retained in addition to GFP-positive cells to serve as untransduced controls. Cells were expanded to passage 2 and 3 following sorting by FACS and plated into Medium A or Medium C for studies of proliferative capacity and neural marker expression. To confirm deletion of Notch 1 by lentiviral-Cre, we performed real-time QPCR for Notch 1; the C_t_ values for Notch 1 mRNA in cultures treated with lentiviral-Cre were >32.7. The relative cell density in mouse ADAS cell cultures treated with lenti-Cre, lenti-mutant Cre, or no lentivirus was the same after 3 days in culture **(** **Figure 4C–E** **)**. Quantitative analysis indicated that deletion of Notch 1 *in vitro* did not significantly affect the proliferative capacity or clonogenic potential of ADAS cells *in vitro* **(** **Figure 4F** **)**, indicating that endogenous Notch 1 does not play a signaling role in the regulation of ADAS cell growth and proliferation.

**Figure 5:**
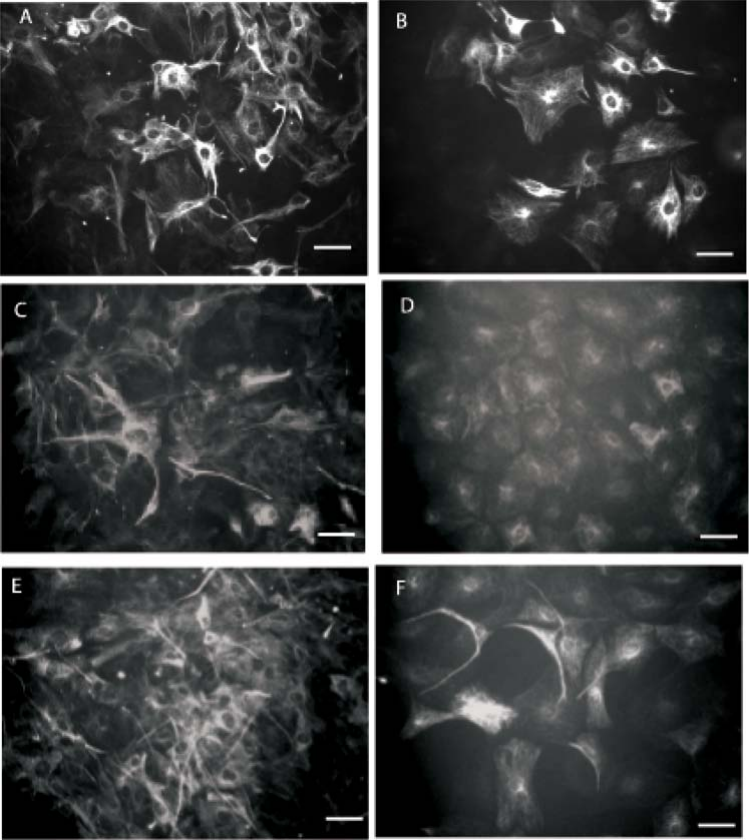
Deletion of Notch 1 does not influence neural marker expression in mouse ADAS cells. Nestin expression in floxed Notch 1 ADAS cells in mock-transduced (A, B), transduced with lentiviral-Cre (C, D), or lentiviral-mutant Cre (E, F) in MesenCult (A, C, E) was decreased by 70% after exposure to NeuroCult Differentiation Medium (B, D, F). as measured by optical density analyses (See Methods). Scale bar in A–F = 55 um.

To extend these observations, we investigated the effect of Notch 1 deletion on expression of Nestin, GFAP, NSE, O4 and NG2. Immunocytochemical analyses of proliferating mouse ADAS cells revealed detectable levels of Nestin and Tuj1 immunoreactivity prior to 48-hour incubation in Differentiation Media. Deletion of Notch 1 in floxed Notch 1 ADAS cells *in vitro* after transduction with lentiviral-derived Cre had no effect on Nestin **(** **Figure 5** **)** or Tuj1 (data not shown) expression. In agreement with other experiments **(Supplemental Table S1)** incubation in NeuroCult Differentiation Medium for 48-hrs resulted in approximately 70% downregulation of Nestin expression; deletion of Notch 1 did not augment or attenuate this response **(** **Figure 5** **)**. NG2 expression was not detectable under any experimental condition. Similarly, GFAP expression was low under basal conditions and slightly increased with exposure to NeuroCult Differentiation Medium; deletion of Notch 1 had no effect on expression (data not shown). Overall, Notch 1 deletion by lentiviral-Cre recombinase did not affect ADAS cell expression of neuroglial markers.

We also investigated whether activation of Notch signaling could alter growthor differentiation responses of ADAS cells. Co-culture of rat ADAS cells with an L fibroblast cell line that stably expressed the Notch ligand Jagged 1 resulted in ADAS proliferation responses and cellular marker expression that were indistinguishable from those induced by the parental L fibroblast line (data not shown). To confirm the lack of effect of Notch signaling activation on ADAS cell growth or differentiation, we aimed to investigate whether constitutive activation of Notch signaling altered the differentiation response of ADAS cells to neural induction cocktails [1], [2]. Rat ADAS cells were nucleofected with a plasmid encoding the Notch intracellular domain (NICD), or with a plasmid encoding GFP, or with no vector at all. Although expression of GFP was detectable by green fluorescence in live cells and expression of NICD could be confirmed by immunoblot analysis (data not shown), ADAS cell growth and morphology was the same under all conditions. Moreover, we were unable to determine whether constitutive activation of the Notch signaling pathway could alter the differentiation response to neuritizing cocktails because nucleofection was not well tolerated by ADAS cells. Specifically, none of the electroporated cells displayed the expected differentiation response to neuritizing cocktails (data not shown). In summary, neither deletion of endogenous Notch 1 nor constitutive activation of the Notch signaling pathway by exogenous ligands or by overexpression of NICD resulted in detectable alteration of ADAS cell morphology, growth characteristics, or cellular marker expression. Taken together, our findings do not support a role for Notch 1 in regulating proliferation or expression of neuro-glial markers in ADAS cells *in vitro.*

### Wnt-1 Lineage Tracing Analysis of mouse ADAS cells

**Figure 6:**
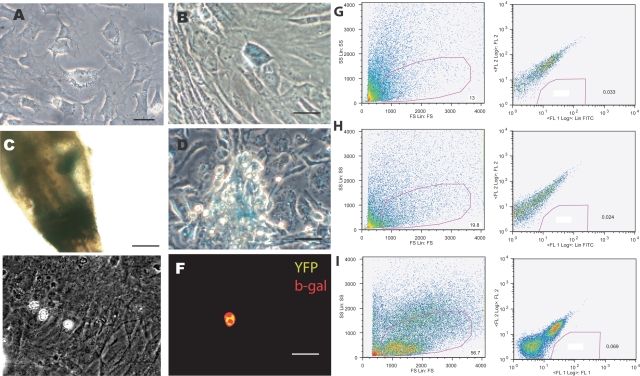
Wnt-1 Lineage tracing analysis reveals the vast majority of cells present in ADAS cultures are not neural crest-derived. Adipose from Wnt1-Cre;Rosa26R-stop-LacZ compound transgenic mice was harvested to establish ADAS cell cultures. X-gal enzymatic activity revealed no detectable blue cells in ADAS cultures grown in NeuroCult (A); a few isolated cells harboring the lacZ transgene in ADAS cultures grown in Mesencult (B); lacZ-positive neural crest-derived cells in the bulb of a whisker hair follicle of a Wnt1-Cre;Rosa26RlacZ transgenic mouse prior to dissociation (C); lacZ-positive cells migrating out of the bulb sheath at the base of a whisker hair follicle in culture after dissociation (D); Phase image of ADAS cultures grown in Mesencult derived from Wnt1-Cre;Rosa26R-stop-lacZ compound transgenic mice (E); Immunofluorescence analysis with an antibody specific for bacterial β-galactosidase revealed the presence of a minimal number of double-labeled EYFP (yellow)/β-galactosidase (red) cells in these cultures (F), n = 3; Quantitative FACS analyses of ADAS cells derived from three different (G, H, I) Wnt1-Cre;Rosa26R-stop-EYFP compound transgenic mice revealed that less than 2% of the cells were EYFP-positive (F), n = 3. Scale bar in A–E = 55 um.

ADAS cells have been reported to express several molecular markers characteristic of neural crest-derived populations [1], [4], [5], [25] and skin-derived precursors (SKPs), which also have neurogenic potential, have been shown to be derived from neural crest [16], [26]. Therefore, we tested the hypothesis that mouse ADAS cells are a neural crest-derived population in adult adipose. The Wnt/beta-catenin signaling pathway is essential to the development of the neural crest [27]–[31]; and Wnt-1-Cre animals crossed with floxed RosaR26R-stop-lacZ reporter mice have been successfully used in lineage tracing analyses to establish that a specific population is derived from neural crest lineage, as cells in which Wnt1 has been activated are indelibly marked and can be traced by the presence of the lacZ transgene [14], [16]. Mouse ADAS cell cultures from adipose harvested from 6- to 9-month old Wnt1–Cre;RosaR26R-lacZ compound transgenic mice were grown in Neurocult or Mesencult then subjected to X-gal enzymatic assays or anti-β galactosidase immunocytochemistry. Hair follicles derived from the whisker barrel of the same transgenic mice were used as positive controls for the presence of the lacZ transgene in the bulb sheath **(** **Figure 6C** **)**. No significant differences in gross morphology or proliferation kinetics were observed between ADAS cultures harvested from wild-type, Wnt-1Cre, Rosa26R-lacZ, or the compound transgenic Wnt1-Cre;Rosa26R-lacZ reporter mice. We found no lacZ-positive (blue) cells in ADAS cell cultures grown in NeuroCult **(** **Figure 6A** **)** and only a few positive cells by X-gal reaction **(** **Figure 6B** **)** or β galactosidase immunocytochemistry **(** **Figure 6E, F** **)** in ADAS cell cultures derived from the compound transgenic Wnt1-Cre;Rosa26R-lacZ mice when grown in Mesencult medium, while lacZ-positive cells were easily detectable migrating out of the base of the bulb sheath when the latter was placed *in vitro* **(** **Figure 6D** **)**. To confirm these results, we performed quantitative FACS analysis of EYPF-positive cells from ADAS cell cultures grown in Mesencult derived from Wnt1-Cre;Rosa26R-stop-EYFP compound transgenic mice. In these mice, expression of the Wnt1 locus during development would indelibly mark cells in which it was expressed and these could then be identified by expression of the fluorescent EYFP protein. We found that less than 2% of the cell population in three independent ADAS cultures derived from compound transgenic mice of either gender were EYFP positive **(** **Figure 6G, H, I** **)**. This small percentage of EYFP-positive cells is within the margin of error of detection for the sorting assay and therefore considered to be insignificant. Lastly, we investigated whether ADAS cells expressed detectable levels of transcription factors or genes characteristic of neural crest-derived tissues, including the Sry/HMG box transcription factor Sox9, the cellular retinoic acid-binding protein II gene (CRABP2), and tyrosine hydroxylase (TH). We did not observe robust expression of these genes in mouse ADAS cells grown in Medium A (Neurocult) or Medium C (Mesencult) (data not shown). On the basis of these multiple negative results, we concluded that ADAS cells do not represent a neural crest-derived population residing in adult adipose.

### Functional Analysis of Neurogenic potential of mouse ADAS cells differentiated by Neural Induction Medium

**Figure 7:**
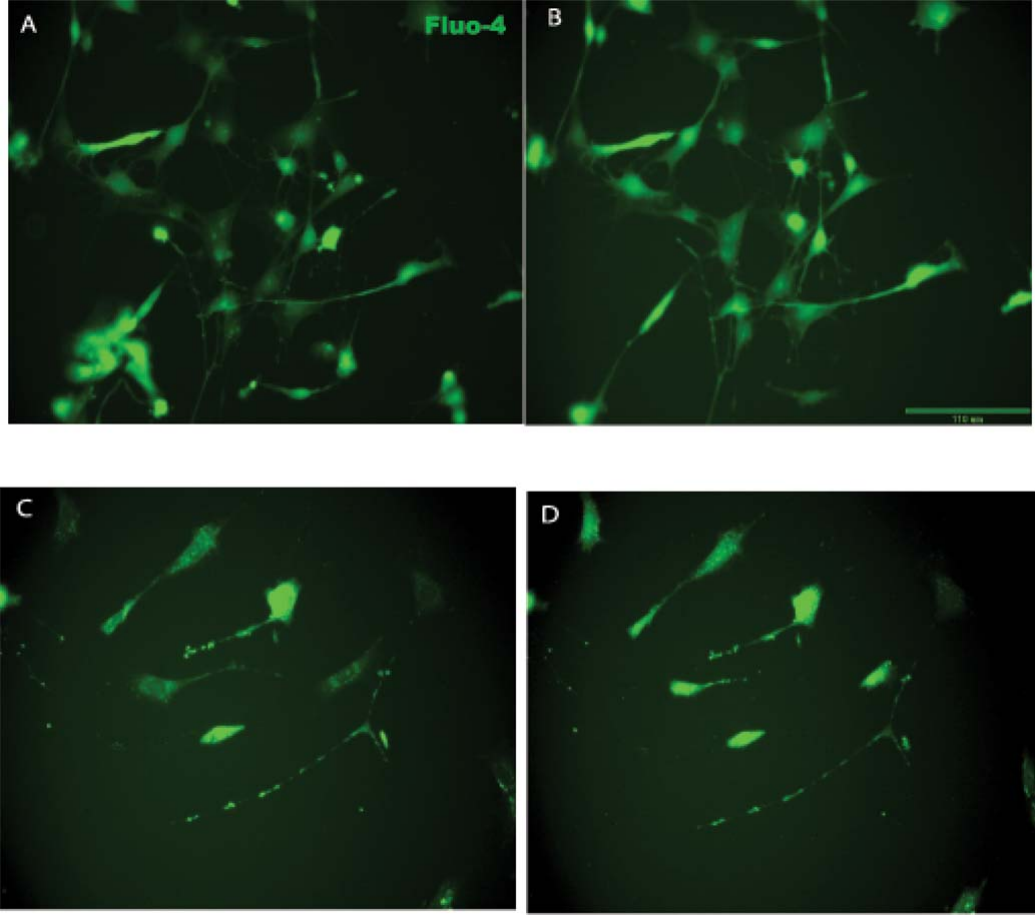
Intracellular calcium imaging in mouse ADAS cells exposed to Neural Induction Medium (NIM). Fluo-4AM fluorescence intensity was monitored in mouse ADAS cells after a 4-hr (A, B) or 24-hr exposure to NIM (C, D), prior to (A, C) or following (B, D) addition of high KCl. Scale bar = 110 um.

We investigated whether exposure of ADAS cells to neural induction cocktails induced differentiation into mature neuronal phenotypes by evaluating cell cycle exit (cessation of Ki67 staining), depolarization-induced calcium transients, and neural network activity in ADAS cells exposed to NIM. Immunocytochemical analysis with an antibody specific for nuclear proliferation antigen Ki67 indicated that a large proportion of the cells grown in MesenCult Basal Medium were highly proliferative. Exposure to NIM induced morphological differentiation and process outgrowth within 4 hrs **(** **Figures 2** **and** **3** **)**, but did not induce a majority of cells to exit the cell cycle by 24 hrs as evidenced by persistent Ki67 staining in the nucleus, indicating that ADAS cells did not undergo terminal differentiation **(Supplemental Figure S1).** Next, we used the Ca^2+^ indicator dye Fluo-4 AM to investigate if a depolarizing stimulus (high extracellular potassium) could induce intracellular Ca^2+^ mobilization in mouse ADAS cells grown in MesenCult and exposed to NIM. Within 30–45 seconds of increasing extracellular potassium to a final concentration of 40 mM, a subset of ADAS cells displayed small but detectable Ca^2+^ transients **(** **Figure 7** **)**, suggesting that a fraction of ADAS cells were excitable and capable of Ca^2+^ mobilization after NIM exposure. To extend these observations we attempted to perform electrophysiological recordings of ADAS cells after NIM exposure. However, efforts to patch these cells were largely unsuccessful (Brad Pfeiffer, personal communication). ADAS cells that could be patched were found to have resting membrane potentials of around -40 mV which are uncharacteristic of most healthy neurons. Similarly, no resting or evoked neural network activity was observed in mouse ADAS cell cultures grown for a number of weeks on Multi-Electrode Arrays (MEAs) **(Supplemental Figure S2)**. Taken together, these data indicate that exposure to neuritizing cocktails induces process outgrowth, expression of neuroglial markers, and membrane excitability in ADAS cells; but electrophysiological findings do not support the claim that ADAS cells grown in media optimized for NSCs or MSCs are competent to differentiate into mature and functional neurons upon exposure to neuritizing stimuli.

## Discussion

Given the ease with which adipose-derived adult stromal (ADAS) cells could be expanded *in vitro* beyond 20 passages while retaining high levels of Nestin expression, it is possible that ADAS cells represent a type of adult mesenchymal stem cell population residing in adipose, but not an adult neural stem cell. Specifically, our studies indicate that ADAS cells expanded and maintained in media formulations optimized for neural stem cells have limited capacity for self-renewal, clonogenicity, and neurosphere formation. Second, although our molecular and cellular analyses of early neural marker expression in ADAS cells after exposure to neural induction cocktails confirm previously published reports [1], [3], we find little physiological evidence to support the claim that the differentiated morphology displayed by ADAS cells after neural induction represents maturation of ADAS cells into functional neurons. Mesenchymal adult stem cell populations from adult human and mouse sources, including bone marrow stromal cells (BMSC) have been reported to have neuron-like characteristics [32], to express neuro-glial specific markers, and to display potential for neuro-glial differentiation both *in vitro* and *in vivo* [33]–[36] but some of the chemical formulations used to induce neural differentiation in these cell types have been called into question [24]. Other reports of *in vitro* neuro-glial differentiation include a population of multipotent adult progenitor cells (MAPCs) that co-purify with mesenchymal stem cells isolated from bone marrow [37].

We find no evidence that Notch 1, a key gatekeeper of progenitor populations and regulator of glial and neural stem cell fates [7], [38], regulates the proliferation, growth, or differentiation-induced expression of neural or glial markers in mouse ADAS cells, arguing strongly against the notion that ADAS cell differentiation into neuron-like cells represent a regulated process analogous to what occurs during differentiation of neuro-glial progenitors. In fact, our findings are consistent with the observation that Notch 1 is dispensable for adipocyte specification and differentiation from either mesenchymal or epithelial progenitors [39]. Although reports indicate that constitutive Notch 1 activation in bone marrow stromal cells exposed to FGF-2, forskolin, and ciliary neurotrophic factor promoted neuronal induction without glial differentiation [40], our findings on genetic ablation of Notch 1 indicate that endogenous Notch 1 signaling does not regulate proliferation or expression of neuro-glial markers in ADAS cells.

Because Notch 1 signaling had been shown to be instructive of secondary fates in neural crest cells [10], [13], we pursued in parallel to Notch deletion experiments the possibility that ADAS cells represented a neural crest population residing in adult adipose. However, Wnt-1 lineage tracing analyses indicated that ADAS cells are not of neural crest origin, in contrast to what has been reported for skin-derived precursor cells (SKPs) which also display neurogenic potential *in vitro* [16]. One key fact that could account for these differences is that SKPs reside in epidermis, an ectodermally-derived tissue.

Taken together, these critical findings do not support the claim that the neurogenic potential of ADAS cells can be attributed to them being adult neural stem cells. Given that co-culture with astrocytes is critical for inducing robust neuronal fates in adult neural stem cells *in vitro* [41], we cannot rule out the possibility that conditions not examined by our experimental paradigms may be capable of inducing terminal differentiation and maturation of ADAS cells. More importantly, *in vivo* studies will be needed to determine the extent to which tissue-specific growth, differentiation factors, and cell-to-cell contacts between ADAS cells and endogenous progenitor or stem cell populations could promote ADAS cell engraftment at a lesion site, thereby enhancing the neuro-glial differentiation and maturation of ADAS cells *in situ.* In support of this possibility, both mouse and human ADAS cell populations have been shown to afford functional benefits in ischemia models in part because they secrete detectable levels of anti-apoptotic and pro-angiogenic factors such as vascular endothelial growth factor (VEGF) detectable using real-time quantitative PCR analysis [42]–[44].

In summary, while it is unlikely that ADAS cells will be able to directly replace lost neurons and restore function to neuronal circuits through mechanisms that involve ADAS cells adopting and maintaining robust neuronal phenotypes, ADAS cells may still be able to contribute to neural repair through other mechanisms, including modification of the environment surrounding a lesion by production of trophic factors, anti-oxidants, or matrix metalloproteases. Additional studies are needed to determine the extent to which autologous transplants of ADAS cells at an injury site survive, engraft, and interact with endogenous progenitor populations to enhance neural repair.

## Supporting Information

Figure S1 Neural Induction Medium (NIM) does not induce terminal differentiation of mouse ADAS cells. Immunocytochemical detection of nuclear proliferation antigen Ki67 (A) demonstrates high proliferative activity in mouse ADAS cells grown in Mesencult; nuclei were visualized by bis-benzimide staining (B and E); merged images (C and F). Persistent Ki67 staining in ADAS cells after 24-hr exposure to NIM (C) indicates not all cells have exited the cell cycle.
(10.89 MB EPS)

Figure S2 Neural network activity recordings from mouse primary hippocampal neurons and mouse ADAS cultures grown on multi-electrode arrays (MEAs). Hippocampus cultures elicit spontaneous activity and are activated by electrical stimulation: 30 sec of spontaneous activity recorded simultaneously from a 20 d.i.v. culture at 17 separate electrodes (A). Each row represents activity from 1 neuron, tick marks within each row demarcate the time the neuron fired an action potential (25 µsec resolution). Electrically evoked activity from a hippocampal culture recorded from one electrode. 10 Hz, 10 µA stimulus marked by red vertical bars, neural responses to stimulus in black (B); No basal or evoked neural network activity was detectable in mouse ADAS cultures grown in Neurocult or Mesencult (C, D); phase images taken with a 4× (E) and 10× objective (F) of mouse ADAS cultures grown on MEAs in Mesencult.
(1.53 MB EPS)

Table S1 Expression of Neural Stem Cell markers in mouse ADAS grown under defined media conditions. Mouse ADAS cells were grown in Media A, B or C for 48 hrs then fixed in 4% paraformaldehyde for immunocytochemical analyses or exposed for an additional 48 hrs to Neurocult differentiation supplements (in the case of Media A and B) or to NIM (in the case of Media C). Semi-quantitative assessment of marker expression was performed using the following criteria: +++ = robust expression in greater than 50% of the cells; ++ = moderate expression in greater than 50% of the cells; + = low expression in less than 50% of the cells; − = no detectable expression. Abbreviations: NSC, Neural Stem Cell; NSE, Neuron Specific Enolase; GFAP, Glial fibrillary acidic protein; Tuj1, β-tubulin III.
(0.03 MB DOC)
