# Supplementary material for: The Neuro-Glial Properties of Adipose-Derived Adult Stromal (ADAS) Cells Are Not Regulated by Notch 1 and Are Not Derived from Neural Crest Lineage
Source: PLoS One. 2008 Jan 16;3(1):e1453. doi: 10.1371/journal.pone.0001453 (PMC2180194; doi:10.1371/journal.pone.0001453)
Supplement: Table S1 — Expression of Neural Stem Cell markers in mouse ADAS grown under defined media conditions. Mouse ADAS cells were grown in Media A, B or C for 48 hrs then fixed in 4% paraformaldehyde for immunocytochemical analyses or exposed for an additional 48 hrs to Neurocult differentiation supplements (in the case of Media A and B) or to NIM (in the case of Media C). Semi-quantitative assessment of marker expression was performed using the following criteria: +++ = robust expression in greater than 50% of the cells; ++ = moderate expression in greater than 50% of the cells; + = low expression in less than 50% of the cells; − = no detectable expression. Abbreviations: NSC, Neural Stem Cell; NSE, Neuron Specific Enolase; GFAP, Glial fibrillary acidic protein; Tuj1, β-tubulin III. (0.03 MB DOC) [file pone.0001453.s003.doc]

**Supplemental Table S1. Expression of Neural Stem Cell markers in Mouse ADAS grown**

**under defined media conditions**

| **Treatment** | **Nestin** | **NSE** | **GFAP** | **Tuj1** |
| --- | --- | --- | --- | --- |
| Medium A:  Serum-free NSC medium | ++ | +++ | +++ | ++ |
| Medium B:  Neurocult medium | ++ | +++ | +++ | ++ |
| Medium C:  Mesencult medium | ++ | +/- | +/- | + |
| Media A or Bà  Neurocult Differentiation  Supplements 48 hrs | + | ++ | +++ | + |
| Medium C 🡪 NIM 48 hrs | + | +++ | ++ | ++ |
